# Occurrence and Characterization of *Cronobacter* spp. in Dehydrated Rice Powder from Chinese Supermarket

**DOI:** 10.1371/journal.pone.0131053

**Published:** 2015-07-01

**Authors:** Yan Huang, Yiheng Pang, Hong Wang, Zhengzhu Tang, Yan Zhou, Weiyu Zhang, Xiugui Li, Dongmei Tan, Jian Li, Ying Lin, Xiaoling Liu, Weiyi Huang, Yunliang Shi

**Affiliations:** 1 Guangxi Zhuang Autonomous Region Center for Disease Control and Prevention, Nanning, Guangxi, China; 2 The First Affiliated Hospital of Guangxi Medical University, Nanning, Guangxi, China; 3 Food Quality and Safety Center of Guangxi University, Nanning, Guangxi, China; 4 College of Animal Science and Technology, Guangxi University, Nanning, Guangxi, China; The University of Hong Kong, HONG KONG

## Abstract

*Cronobacter* spp. are emerging food-borne pathogens and have been identified as causative agents of meningitis and necrotizing enterocolitis in infants. Dehydrated rice is popular with a wide range of people and it is frequently used as a substitute for infant milk powder to baby older than four months. The occurrence of *Cronobacter* spp. was investigated in 1,012 samples of dehydrated rice powder collected from 14 manufacturers in China during 2010 to 2012. The isolates were identified using fusA allele sequencing and subtyped using pulsed-field gel electrophoresis. Seventy-six samples (7.5%) contained *Cronobacter* spp. The prevalence among manufacturers ranged from 0-28.8%. The 76 isolates included 4 species [*Cronobacter sakazakii* (52 isolates) *Cronobacter malonaticus* (14 isolates), *Cronobacter dublinensis* (7 isolates), and *Cronobacter muytjensii* (3 isolates)]. Twenty-three unique fusA alleles and sixty-six PFGE-patterns were detected. All isolated strains were observed to be sensitive or to show intermediate susceptibility to eight tested antimicrobial agents. The study revealed serious contamination of dehydrated rice powder by *Cronobacter* spp., with prevalence varying among manufacturers in China. Identified *Cronobacter* species, fusA alleles, and subtypes were diverse.

## Introduction

The bacterium *Enterobacter sakazakii*, was transferred a new genus *Cronobacter* in 2007 [1]. Six other species have now been identified: *Cronobacter malonaticus*, *Cronobacter universalis*, *Cronobacter turicensis*, *Cronobacter muytjensii*, *Cronobacter dublinensis* and *Cronobacter condimenti* [2–3]. All seven species are recognized as emerging opportunistic food-borne pathogens associated with human disease [4–5] where they infect all age groups [5–6]. Neonates, especially those premature or with low birth weight, are at highest risk for *Cronobacter* infection which causes meningitis and necrotizing enterocolitis [7], resulting in mortality rates of 10%–80% [5, 8–12]. Infections in adults may cause conjunctivitis, biliary sepsis, urosepsis, appendicitis, wound infections and pneumonia in vulnerable adults who are more susceptible and at increased risk of infection [13].

*Cronobacter* spp. have been isolated from food and environmental, human, and clinical sources. A wide variety of foods including infant formula, cheese, dried foods, meats, and plant materials, as well as water can be contaminated with *Cronobacter* [14–20]. Dehydrated rice powder (DRP) is a good substitute for powdered infant formula (PIF) and is popular with a broad range of the population, but rice powder products have been found to be contaminated with *Cronobacter* [21–22]. Infant rice cereal reconstituted with water, milk, or infant formula supports copious growth of *C*. *sakazakii*, the species most commonly associated with infant disease [13,23]. However, investigation of *Cronobacter* presence in DRP is limited. To determine *Cronobacter* spp. contamination of DRP from manufacturers in China, we examined 1,012 samples from 14 manufacturers during 2010–2012 for the prevalence of *Cronobacter* spp. and the characteristics of the isolates including biochemical character, fusA alleles sequences, pulsed-field gel electrophoresis (PFGE) patterns, and antibiotic susceptibility.

## Materials and Methods

### Ethics statement

1012 DRP samples from 14 manufacturers and was collected from 6 supermarkets. The products that were found to be contaminated of *Cronobacter* in this study have been notified of the results to the manufacturers/supermarkets. On the request of the DRP manufacturers, the brand names and catalog/lot number of the DRP did not publishing but appear anonymously.

### Sample collection

Sealed packages of DRP (n = 1,012) from 14 food manufacturers were purchased in 2010 to 2012 from different supermarkets in China (Table 1). These products contained additional small amount of ingredients such as zinc, Chinese yam powder, carrot powder and dried fruit for increased nutrition. The samples were immediately taken to the laboratory and examined for the presence of *Cronobacter* spp. under aseptic conditions on the day of arrival.

**Table 1:** *Cronobacter* spp. isolated from dehydrated rice powder (DRP) detected by culture and 16S rRNA gene PCR amplification and sequencing.

Manufacturer	Foods	Samples (n)	Positive by culture(%)	16S PCR and sequencing (%)
A	DRP+ Chinese yam powder	132	38(28.8)	38(28.8)
B	DRP	124	9(7.3)	9(7.3)
C	DRP	120	4(3.3)	4(3.3)
D	DRP	106	4(3.8)	4(3.8)
E	DRP	103	5(4.9)	5(4.9)
F	DRP	91	3(3.3)	3(3.3)
G	DRP	84	4(4.8)	4(4.8)
H	DRP+ Chinese yam powder	47	5(10.6)	5(10.6)
I	DRP	45	1(2.2)	1(2.2)
J	DRP	42	0(0.0)	0(0.0)
K	DRP	40	1(2.5)	1(2.5)
L	DRP	38	0(0.0)	0(0.0)
M	DRP	22	1(4.5)	1(4.5)
N	DRP	18	1(5.6)	1(5.6)
Total		1012	76(7.5)	76(7.5)

### Isolation and identification of *Cronobacter* spp.

The procedure adopted for the isolation of *Cronobacter* spp. was based on Iversen (2007) [24] and the original *Enterobacter sakazakii* technical specifications (ISO/TS 22964, 2006). Briefly, a 100 g DRP sample was homogenized with 900 mL buffered peptone water (BPW; OXOID, Hampshire, UK) and incubated at 37°C for 18 hr as a pre-enrichment step. Subsequently, 1 mL of the BPW suspension was transferred to 10 mL modified lauryl sulfate tryptose broth (OXOID, Hampshire, UK), and after further incubation at 42°C for 24 hr, the broth was streaked on Druggan Forsythe Iversen (DFI, Oxoid, Hampshire, UK) agar and incubated at 37°C for 24 hr. Greenish/blue colonies on DFI agar were considered presumptive *Cronobacter* spp. The suspected isolates were subjected to the biochemical galleries by VITEK 2 compact GN (bioMèrieix, France) for identification of *Cronobacter*, according to the manufacturer's instructions. Two reference strains *Cronobacter muytjensii* ATCC51329 and *Cronobacter sakazakii* ATCC 25944 purchased from American Type Culture Collection. Supplemental biochemical differentiation tests were performed according to Farmer (1980) and Iversen (2006) [25–26].

### DNA extraction

Presumptive Cronobacter isolates were incubated overnight at 37°C in Luria Bertani broth. Genomic DNA was extracted using a Genomic DNA isolation kit (Qiagen, Germany) according to the manufacturer’s instructions. Concentration and purity of DNA samples were estimated by Nanodrop2000 (Thermo Fisher Scientific, USA).

### 16S rRNA gene sequences amplification and sequencing

DNA sequencing for the 16S rRNA segment was performed as described by Iversen et al. [1]. PCR amplification using the primers P0 (5'-AGA GTT TGA TCC TGG CTC AG-3') and P6 (5'-GTACGG CTA CCT TGT TAC GA-3'). PCR reaction mixtures were conducted on a volume of 50 μL composed of 1×PCR buffer, 1× Q solution, 2 mM MgCl2, 0.25 mM dNTPs, 1 pM of each primer, 1 U Taq polymerase (Qiagen, UK), and ~10ng chromosomal DNA with conditions: 3 min at 95°C; 30 cycles of 30 sec at 95°C, 30 sec at 54°C, and 2 min at 72°C; and a final extension of 5 min at 72°C. The PCR products were cut from 1% gel visualized under UV. After purification the amplified fragments were double stranded sequencing and triple repeats using the primers P0 and P6 by DNA analyzer (ABI 3730xl; USA). All 16S rRNA gene sequences of the 76 isolated strains were submitted to GenBank.

### fusA gene sequences amplification and sequencing

The fusA gene PCR amplification was performed as described in Baldwin et al. [27]. After purification using a PCR purification Kit (Qiagen, Maryland, US) according to the manufacturer’s instructions, the amplified fragments were double stranded sequencing and triple repeats using the sequencing primers by DNA analyzer (ABI 3730xl; USA). The fusA sequences were queried in MLST databases http://pubmlst.org/cronobacter/info/protocol.shtml to identify the species and fusA alleles sequences. A phylogenetic tree was constructed based on the sequences of the fusA alleles (438 bp) using the maximum likelihood algorithm in MEGA 6 (v. 6.06) [28].

### PFGE of the isolates

Pulsed-field gel electrophoresis (PFGE) of *Cronobacter* isolates was performed as described by Brengi et al. [29]. Strains were grown overnight on blood agar plates and were harvested. XbaI was used as primary restriction enzyme. DNA fragments were separated by electrophoresis (CHEF Mapper, Bio-Rad Laboratories, Hercules, California, US) through a 1% (w/v) agarose gel (Seakem Gold, Rockland, Maine, US) in 0.5×TBE buffer using the program: initial switch time of 1.8 s and final switch time of 25 sec, 20 hr, 120° angle at 6 volts/cm. Gels were stained with GelRed (Biotium, CA, US) and visualized under U.V. light using a GelDoc XR+ system (Bio-Rad laboratories, Hercules, California, US). Dendrograms were constructed using BioNumerics v. 6.6 (Applied Maths, Sint-Martens-Latem, Belgium) and cluster analysis conducted using the DICE coefficient and unweighted pair group method with arithmetic means (UPGMA). Band position tolerance and optimization coefficient were 1.5%. A *Salmonella* serotype Braenderup strain (H9812) was chosen as the universal size standard.

### Antimicrobial susceptibility testing

All isolates were tested using the broth microdilution method as described by the Clinical and Laboratory Standards (CLSI, M7-A7, 2006) [30] for susceptibility to 8 antimicrobial agents (Table 2). *Escherichia coli* ATCC 25922 was used as reference stain. The minimal inhibitory concentrations (MIC) were read manually using a light box for interpretation in accordance with criteria provided by CLSI, 2010 [31].

**Table 2:** Susceptibility of *Cronobacter* isolates in dehydrated rice powder samples (n = 76) to antimicrobial agents.

Antibiotics	No. of isolates (%)		
	Susceptible	Intermediate	Resistant
Chloramphenicol	72(94.7)	4(5.3)	0(0)
Trimethoprim/sulfamethoxazole	76(100)	0(0)	0(0)
Gentamicin	76(100)	0(0)	0(0)
Cefotaxime	76(100)	0(0)	0(0)
Cefoxitin	68(89.5)	8(10.5)	0(0)
Nalidixic acid	76(100)	0(0)	0(0)
Ciprofloxacin	76(100)	0(0)	0(0)
Tetracycline	76(100)	0(0)	0(0)

## Results

### Incidence of *Cronobacter* in DRP

Seventy-six of the 1012 tested DRP samples were found positive for *Cronobacter* by culture and 16S rRNA gene PCR amplification and sequencing. The contamination rate among manufacturers ranged from 0–28.8% (Table 1). Two manufacturers (A and H) showed >10% contamination rates, the highest 28.8%. Two manufacturers (J and L) contained no detectable *Cronobacter*, and the contamination rate in the remainder was 2.2–7.3% (Table 1).

### Biochemical analysis

The biochemical testing categorized the 72 strains of *Cronobacter* spp. into 10 biogroups as previously described by Farmer (1980) and Iversen (2006) [25–26] (Table 3). Biogroups 2 (n = 30), 9 (n = 14), and 1 (n = 12) were the main phenotypes; biogroups 8a and 14a each contained a single isolate. The remaining four isolates (WJ1333, WJ1622, WJ1334, and WJ0016) could not be assigned to any original *Cronobacter* spp biochemical types (biogroup 1 to 16) [1, 25–26] and formed three unknown biogroups (Table 3).

**Table 3:** Assignment of strains to biogroups based on Farmer (1980) and Iversen (2006).

Biogroups	Phenotype^a^											No. of strains
	VP	MR	Nit	Orn	Mot	Ino	Dul	Ind	Malo	Gas	AMG	
**1**	+	-	+	+	+	+	-	-	-	+	+	**12**
**2**	+	-	+	+	+	-	-	-	-	+	+	**30**
**5**	+	-	+	+	+	+	-	-	+	+	+	**3**
**6**	+	-	+	+	+	+	-	+	-	+	+	**1**
**8a**	+	-	-	+	+	-	-	-	-	+	+	**1**
**9**	+	-	+	+	+	-	-	-	+	+	+	**14**
**10**	+	-	+	+	+	-	-	+	-	+	+	**6**
**11**	+	-	+	+	+	-	+	-	-	+	+	**4**
**14a**	+	-	+	-	+	-	-	-	-	+	+	**1**
**15**	+	-	+	+	+	+	+	+	+	+	-	**1** ^b^
**Unmatched 1**	+	-	-	+	+	-	-	-	-	-	+	**1**
**Unmatched 2**	+	-	+	+	-	-	+	-	+	+	-	**1**
**Unmatched 3**	+	-	+	+	-	+	+	+	+	+	-	**2**

^a^VP, Voges-Proskauer; MR, methyl red; Nit, nitrate reduction; Orn, ornithine decarboxylation; Mot, motility at 37°C; Ino, acid production from inositol; Dul, acid production from dulcitol; Ind, indole production; Malo, malonate utilization; Gas, gas production from glucose; AMG, acid production for methyl-α-D-glucoside.

^b^C. muytiensii ATCC 51329.

### Species identification

**Fig 1:**
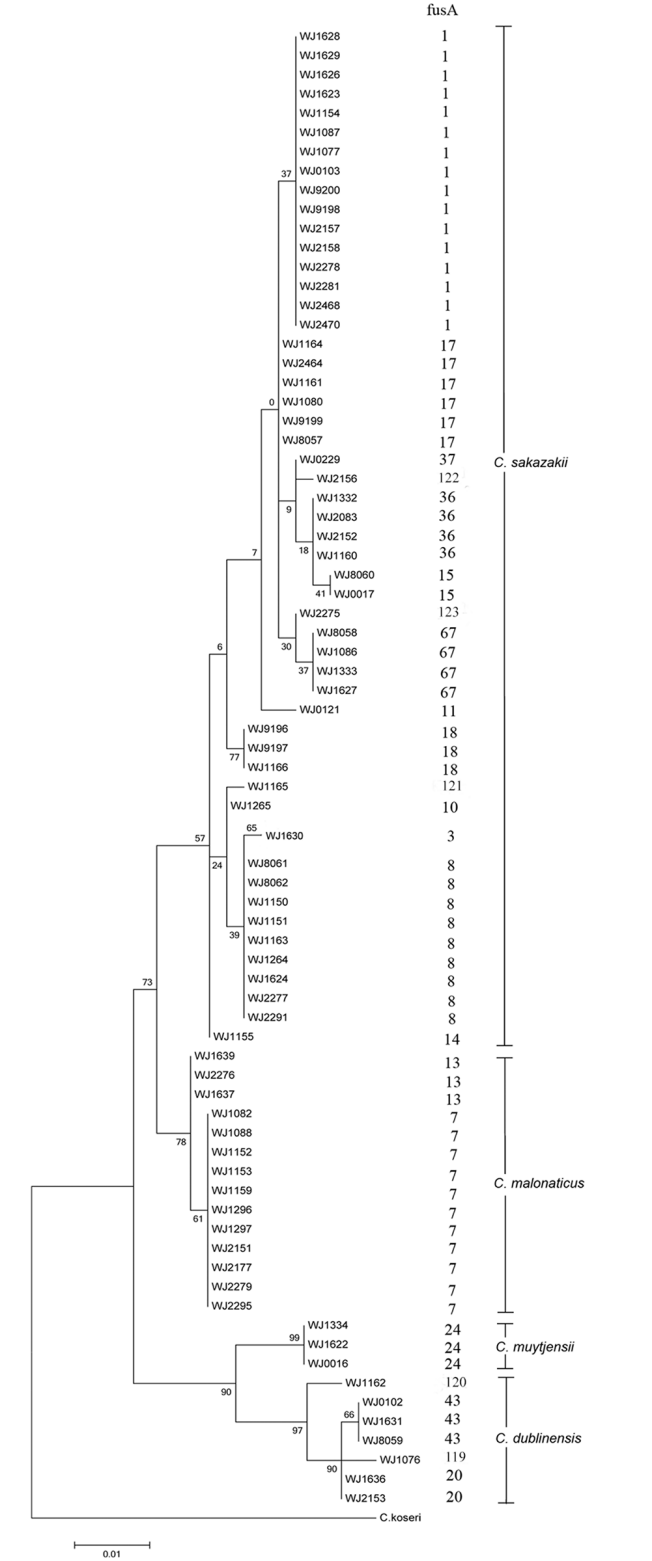
Maximum likelihood tree of the 76 isolated *Cronobacter* and outgroup species *Citrobacter koseri* based on the fusA alleles (438 bp) of the *Cronobacter* multilocus sequence typing dataset. This tree was generated using the MEGA (v. 6.06) with 1000 bootstrap replicates.

**Fig 2:**
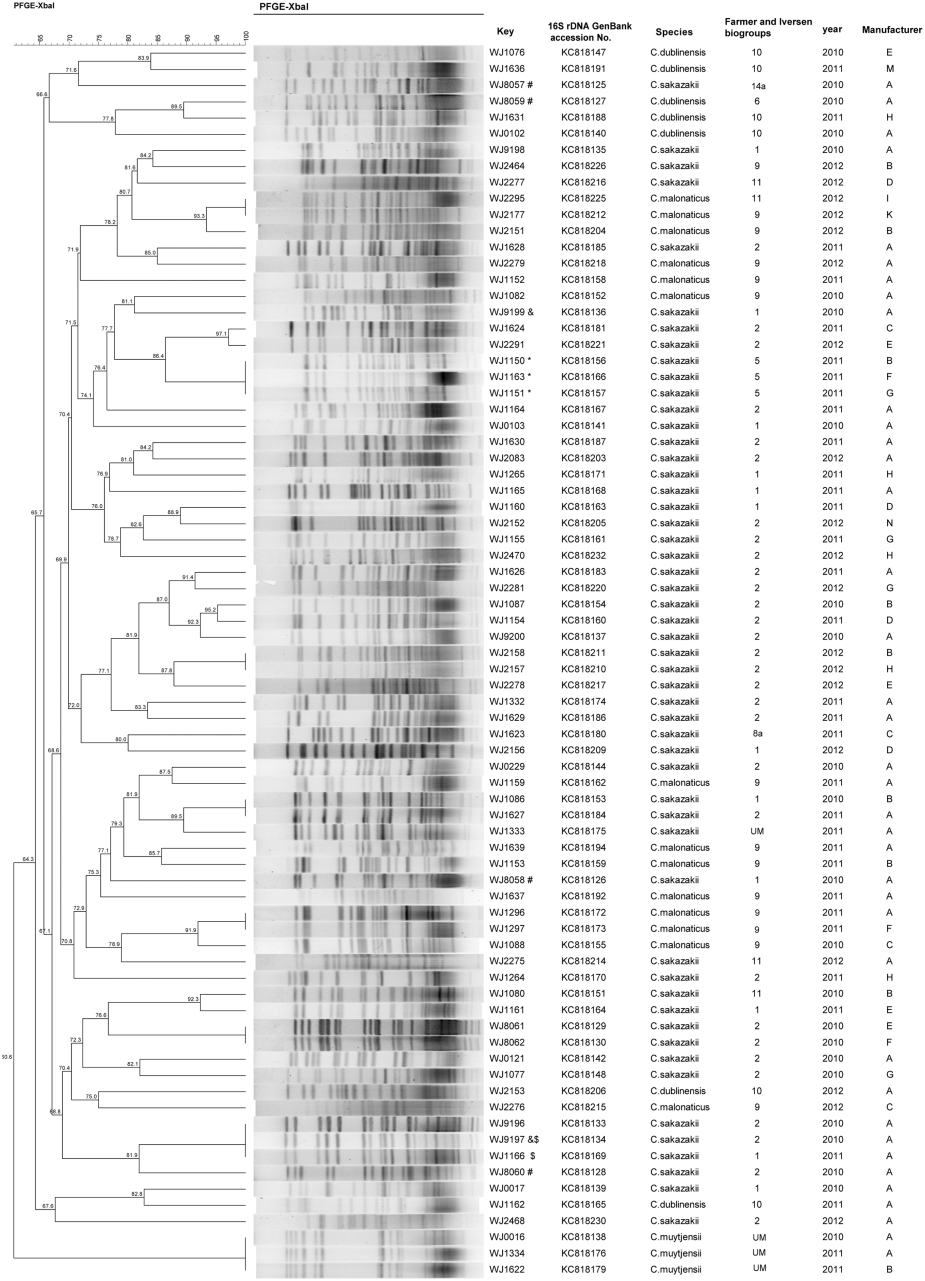
Dendrogram combining PFGE patterns of XbaI-digested DNA from the 76 isolated *Cronobacter* spp. Isolate information including GenBank accession No., biogroups, isolation years, and manufacturer. * Three manufacturers shared the genetic subtypes MJ1150, WJ1151, and WJ1163; & Species isolates from the same batch showed different genetic subtypes (WJ9197 and WJ 9199); # A single batch of rice powder from manufacturer A contained two species (WJ0857, WJ0858, and WJ 8060—*C*. *sakazakii*; WJ8059—*C*. *dublinensis*); $ the same PFGE-pattern in batches produced a year apart, implying ongoing contamination of the rice product (WJ9197 and WJ1166).

The 76 isolates *of Cronobacter* spp. were identified as four species based on the fusA allele in Joseph’s study [2]: *C*. *sakazakii* (52 isolates), *C*. *malonaticus* (14 isolates), *C*. *dublinensis* (7 isolates), and *C*. *muytjensii* (3 isolates) (Fig 1). Eighteen fusA alleles (1, 2, 3, 7, 8, 10, 11, 13, 14, 15, 17, 18, 20, 24, 36, 37, 43, and 67) were identified using the MLST database, five unmatched sequences were sent to MLST manager and generated five new allele fusA sequences (fusA 119 to 123) (Fig 1). A phylogenetic tree was constructed based on the fusA sequences of the 76 isolates, and no isolates were shared among the four *Cronobacter* species (Fig 1). Seventy-six nearly complete 16S rDNA sequences (~1.5 kb) were obtained, submitted to GenBank database, and assigned accession numbers (Fig 2).

### PFGE

BioNumerics software analysis showed the 76 isolates to demonstrate 66 distinguishable Xba I PFGE patterns (Fig 2). A high degree of genetic diversity was revealed using PFGE after genomic DNA digestion; the discriminatory index was 0.9940, as calculated using Simpson’s diversity index. Within the 66 PFGE-patterns, *C*. *sakazakii*, *C*. *malonaticus*, and *C*. *dublinensis* exhibited high diversity, while *C*. *muytjensii* showed a common pattern (Fig 2).

### Antibiotic susceptibility

The resistance pattern of the 76 *Cronobacter* isolate strains to the 8 antimicrobial agents is shown in Table 2. All natural isolates were found susceptible to trimethoprim/sulfamethoxazole, gentamicin, cefotaxime, nalidixic acid, ciprofloxacin and tetracycline. Eight (10.5%) and four isolates (5.3%) showed only intermediate resistance to cefoxitin and chloramphenicol, respectively.

## Discussion

Great attention has been focused on the contamination of PIF by *Cronobacter* spp. [32–34], while information concerning such contamination of DRP products is limited. In the present study, 1012 samples of DRP products from fourteen manufacturers were investigated for the presence of *Cronobacter* in 2010–2012. *Cronobacter* spp. contamination was revealed to be serious, occurring at an overall infection rate of 7.5%, with the highest level of contamination being 28.8%, much greater than seen in PIF (2–22%) [7].

Contamination rate varied widely among manufacturers. Some brands contained additional ingredients such as Chinese yam powder and dried fruit for increased nutrition. These ingredients added to rice powder without heat may increase the risk of contamination [35]. In our study, two manufacturers (A and H) that showed a high contamination rate were supplemented with Chinese yam powder, which may have been a contributing factor. Criteria regulating *Cronobacter* spp. have been established for infant formulas with an intended target age <6 months [36], and European Commission Regulation (No. 2073/2005; No. 1441/2007) requires a zero tolerance of *Cronobacter* presence in powdered formula intended for infants under 6 months of age. However, there is less attention and no established criteria for DRP *Cronobacter* contamination, although these foods are frequently used as a substitute for PIF to infant older than four months in some less affluent regions.

The previous study demonstrated that the 16S rRNA gene and the biotyping is not suitable for *Cronobacter* species identification [27]. Multilocus sequence typing (MLST) approach employing the alleles of 7 genes (atpD, fusA, glnS, gltB, gyrB, infB, and ppsA) showed can identify the *Cronobacter* species. Among the seven loci, fusA was observed to be the least number of shared alleles among species, and none of the profiles were shared between two or more species is adequate for speciation [2]. Based on fusA sequencing and analysis, we identified four species of *Cronobacter* from DRP. *C*. *sakazakii* and *C*. *malonaticus* to be the most common species (66/76), similar to results of other studies [37–38]. And the two species most commonly associated with human disease [13]. Products of one manufacturer contained four *Cronobacter* species. More than one species was detected in products of six manufacturers, and different species were isolated from the same batch. A single batch of DRP from manufacturer A contained both *C*. *sakazakii* and *C*. *dublinensis* (Fig 2), indicating that mixed contamination is possible and stresses the need to examine more than one colony from each batch of rice powder.

PFGE is well-established and widely used for the molecular subtyping of bacteria, including *Cronobacter* spp. [39–41]. Sixty-six PFGE patterns were observed among the 76 isolates. Species isolates in the same batch sometimes showed different genetic subtypes (Fig 2), indicating that contamination could have originated from different sources during the manufacturing process (Fig 2). Several manufacturers shared the same genetic subtypes (Fig 2), implying a common original source of contamination. We also observed the same PFGE-pattern in batches produced a year apart, implying ongoing contamination of the rice product (Fig 2). In manufacturer A, 38 isolated *Cronobacter* strains included four species, 14 allele fusA sequences (Fig 2), and 35 PFGE-patterns, revealing that *Cronobacter* contamination of a DRP product is complex and may originate from different sources, including harvest, transport, storage of the rice and the manufacturing process. The prevention of DRP *Cronobacter* contamination not only preventing the rice powder and ingredients contamination, but also get rid of the *Cronobacter* during manufacturing process.

In the biochemical test, three previously unreported biogroups were observed. Recently, new biogroups of *Cronobacter* have been identified [34]. It seems likely that further biogroups remain to be identified. In the antimicrobial susceptibility test, all natural *Cronobacter* isolates were susceptible or intermediately susceptible to chloramphenicol, trimethoprim/sulfamethoxazole, gentamicin, cefotaxime, nalidixic acid, ciprofloxacin, and tetracycline. *Cronobacter* spp. tend to be more sensitive to most antibiotics than other *Enterobacteriaceae*, although resistance to ampicillin and cephalothin resistance has developed [22, 42–44].

## Conclusions

*Cronobacter* contamination of DRP products is a serious concern, with the contamination rate varying among manufacturers. The species, allele fusA sequences, and PFGE patterns of the identified *Cronobacter* isolates from DRP were diverse. The presence of these foodborne pathogens in rice powder products is a potential threat to human health, particularly for infants under 6 months of age and vulnerable adults. Greater attention should be paid to the contamination of DRP.

## References

[1] Iversen C, Lehner A, Mullane N, Bidlas E, Cleenwerck I, Marugg J, et al (2007) The taxonomy of *Enterobacter sakazakii*: proposal of a new genus *Cronobacter* gen. nov. and descriptions of *Cronobacter sakazakii comb*. nov. *Cronobacter* sakazakii subsp. sakazakii, comb. nov., *Cronobacter* sakazak*ii subsp*. *malonaticus* subsp. nov., *Cronobacter turicensis sp*. nov., *Cronobacter muytjensii sp*. nov., *Cronobacter dublinensis sp*. nov. and *Cronobacter* genomospecies 1. BMC Evol Biol 7: 64–84. 17439656 10.1186/1471-2148-7-64 PMC1868726

[2] Joseph S, Sonbol H, Hariri S, Desai P, McClelland M, Forsythe SJ, et al (2012) Diversity of the *Cronobacter* genus as revealed by multi locus sequence typing. J Clin Microbiol 50: 3031–3039. 10.1128/JCM.00905-12 22785185 PMC3421776

[3] Forsythe SJ, Dickins B, Jolley KA (2014) *Cronobacter*, the emergent bacterial pathogen *Enterobacter sakazakii* comes of age; MLST and whole genome sequence analysis. BMC Genomics 15: 1121 10.1186/1471-2164-15-1121 25515150 PMC4377842

[4] Muytjens HL, Zanen HC, Sonderkamp HJ, Kollée LA, Wachsmuth IK, Farmer JJ 3rd, et al (1983) Analysis of eight cases of neonatal meningitis and sepsis due to *Enterobacter sakazakii* . J Clin Microbiol 18: 115–120. 6885983 10.1128/jcm.18.1.115-120.1983 PMC270753

[5] Lai KK (2001) *Enterobacter sakazakii* infections among neonates, infants, children, and adults: case reports and a review of the literature. Medicine 80: 113–122. 11307587 10.1097/00005792-200103000-00004

[6] Nazarowec-White M, Farber JM (1997) *Enterobacter sakazakii*: A review. Int J Food Microbiol 34: 103–113. 9039558 10.1016/s0168-1605(96)01172-5

[7] Caubilla-Barron J, Hurrell E, Townsend S, Cheetham P, Loc-Carrillo C, Fayet O, et al (2007) Genotypic and phenotypic analysis of *Enterobacter sakazakii* strains from an outbreak resulting in fatalities in a neonatal intensive care unit in France. J Clin Microbiol 45: 3979–3985. 17928419 10.1128/JCM.01075-07 PMC2168550

[8] FAO/WHO. 2004. Workshop on *Enterobacter sakazakii* and other microorganisms in powdered infant formula, Geneva, 2–5 February 2004. Available: http://www.who.int/foodsafety/micro/jemra/meetings/feb2004/en/index.html.

[9] FAO/WHO. 2006. Expert meeting on *Enterobacter sakazakii* and *Salmonella* in powdered infant formula, Rome, 16–20 January 2006. Available: http://www.who.int/foodsafety/micro/jemra/meetings/jan2006/en/index.html.

[10] FAO/WHO. 2008. *Enterobacter sakazakii* (*Cronobacter* spp.) in powdered follow-up formulae. Microbiological Risk Assessment Series no. 15. Available: http://www.who.int/foodsafety/publications/micro/mra_followup/en/.

[11] Corti G, Panunzi I, Losco M, Buzzi R (2007) Postsurgical osteomyelitis caused by *Enterobacter sakazakii* in a healthy young man. J Chemother 19: 94–96. 17309858 10.1179/joc.2007.19.1.94

[12] Forsythe SJ (2005) *Enterobacter sakazakii* and other bacteria in powdered infant milk formula. Matern Child Nutr 1: 44–50. 16881878 10.1111/j.1740-8709.2004.00008.x PMC6874386

[13] Holý O. and Forsythe S. (2014) *Cronobacter* spp. as emerging causes of healthcare-associated infection. J Hosp Infect 86: 169–177. 10.1016/j.jhin.2013.09.011 24332367

[14] Muytjens HL, Roelofs-Willemse H, Jaspar GH (1988) Quality of powdered substitutes for breast milk with regard to members of the family Enterobacteriaceae. J Clin Microbiol 26: 743–746. 3284901 10.1128/jcm.26.4.743-746.1988 PMC266435

[15] Biering G, Karlsson S, Clark NC, Jónsdóttir KE, Lúdvígsson P, Steingrimsson O. (1989) Three cases of neonatal meningitis caused by *Enterobacter sakazakii* in powdered milk. J Clin Microbiol 27: 2054–2056. 2778070 10.1128/jcm.27.9.2054-2056.1989 PMC267737

[16] Agostoni C, Axelsson I, Goulet O, Koletzko B, Michaelsen KF, Puntis JW, et al (2004) Preparation and handling of powdered infant formula: a commentary by the ESPGHAN committee on nutrition. J Pediatr Gastroenterol Nutr 39: 320–322. 15448416 10.1097/00005176-200410000-00002

[17] Chap J, Jackson P, Siqueira R, Gaspar N, Quintas C, Park J, et al (2009) International survey of *Cronobacter sakazakii* and other *Cronobacter* spp. in follow up formulas and other Infant foods. Int J Food Microbiol 136: 185–188. 10.1016/j.ijfoodmicro.2009.08.005 19729216

[18] Friedemann M (2007) *Enterobacter sakazakii* in food and beverages (other than infant formula and milk powder). Int J Food Microbiol 116: 1–10. 17331606 10.1016/j.ijfoodmicro.2006.12.018

[19] Baumgartner A, Grand M, Liniger M, Iversen C (2009) Detection and frequency of *Cronobacter* spp. (*Enterobacter sakazakii*) in different categories of ready-to-eat foods other than infant formula. Int J Food Microbiol 136: 189–192. 10.1016/j.ijfoodmicro.2009.04.009 19419789

[20] Healy B, Cooney S, O'Brien S, Iversen C, Whyte P, Nally J, et al (2010) *Cronobacter* (*Enterobacter sakazakii*): an opportunistic foodborne pathogen. Foodborne Pathog Dis 7: 339–350. 10.1089/fpd.2009.0379 19958103

[21] Iversen C. and Forsythe S (2004) Isolation of *Enterobacter sakazakii* and other Enterobacteriaceae from powdered infant formula milk and related products. Food Microbiol 21: 771–777.

[22] Kim K, Jang SS, Kim SK, Park JH, Heu S, Ryu S (2008) Prevalence and genetic diversity of *Enterobacter sakazakii* in ingredients of infant foods. Int J Food Microbiol 122: 196–203. 10.1016/j.ijfoodmicro.2007.11.072 18177966

[23] Richards GM, Gurtler JB, Beuchat LR (2005) Survival and growth of *Enterobacter sakazakii* in infant rice cereal reconstituted with water, milk, liquid infant formula, or apple juice. J Appl Microbiol 99: 844–850. 16162235 10.1111/j.1365-2672.2005.02656.x

[24] Iversen C, Forsythe SJ (2007) Comparison of media for the isolation of *Enterobacter sakazakii* . Appl Environ Microbiol 73: 48–52. 17071794 10.1128/AEM.01562-06 PMC1797115

[25] Farmer JJ, Asbury MA, Hickman FW, Don J and the Enterobacteriaceae study group (1980) *Enterobacter sakazakii*: A New Species of “Enterobacteriaceae” Isolated from Clinical Specimens. Soc General Microbiol 30: 569–584.

[26] Iversen C, Waddington M, Farmer JJ, Forsythe SJ (2006) The biochemical differentiation of *Enterobacter sakazakii* genotypes. BMC Microbiol 6: 94–100. 17067387 10.1186/1471-2180-6-94 PMC1634753

[27] Baldwin A, Loughlin M, Caubilla-Barron J, Kucerova E, Manning G, Dowson C, et al (2009) Multilocus sequence typing of *Cronobacter sakazakii* and *Cronobacter malonaticus* reveals stable clonal structures with clinical significance which do not correlate with biotypes. BMC Microbiol 9: 223 10.1186/1471-2180-9-223 19852808 PMC2770063

[28] Tamura K, Stecher G, Peterson D, Filipski A, Kumar S (2013) MEGA6: Molecular Evolutionary Genetics Analysis version 6.0. Mol Biol Evol 30: 2725–2729 10.1093/molbev/mst197 24132122 PMC3840312

[29] Brengi SP, O'Brien SB, Pichel M, Iversen C, Arduino M, Binsztein N, et al (2012) Development and validation of a PulseNet standardized protocol for subtyping isolates of Cronobacter species: Foodborne Pathog Dis 9: 861–867. 10.1089/fpd.2012.1161 22891917

[30] Clinical and Laboratory Standards Institute (2006) Performance standards for antimicrobial susceptibility test. 16th informational supplement 26:M100–S16.

[31] Clinical and Laboratory Standard Institute (2010) Performance Standards for Antimicrobial Susceptibility Testing; 20th Informational Supplement. CLSI document.M100–S20.

[32] Shaker R, Osaili T, Al-Omary W, Jaradat Z, Al-Zuby M (2007) Isolation of *Enterobacter sakazakii* and other *Enterobacter* sp. from food and food production environments. Food Control 18: 1241–1245.

[33] Restaino L, Frampton EW, Lionberg WC, Becker RJ (2006) A Chromogenic Plating Medium for the Isolation and Identification of *Enterobacter sakazakii* from Foods, Food Ingredients, and Environmental Sources. J Food Prot 69: 315–322. 16496571 10.4315/0362-028x-69.2.315

[34] Lee YD, Park JH, Chang H (2012) Detection, antibiotic susceptibility and biofilm formation of *Cronobacter* spp. from various foods in Korea. Food Control 2: 225–230.

[35] Walsh D, Molloy C, Iversen C, Carroll J, Cagney C, Fanning S, et al (2011) Survival characteristics of environmental and clinically derived strains of *Cronobacter sakazakii* in infant milk formula (IMF) and ingredients. J Appl Microbiol. 110: 697–703. 10.1111/j.1365-2672.2010.04921.x 21255207

[36] Codex Alimentarius Commission (2008) Code of hygienic practice for powdered formulae for infants and young children CAC/RCP 66. Italy: Joint FAO/WHO Food Standards Programme.

[37] El-Sharoud WM, O’Brien S, Negredo C, Iversen C, Fanning S, Fanning S, et al (2009) Characterization of *Cronobacter* recovered from dried milk and related products. BMC Microbiol 9: 24 10.1186/1471-2180-9-24 19187534 PMC2640398

[38] Kim JB, Kang SH, Park YB, Choi JH, Park SJ, Cho SH, et al (2011) The phenotypic and genotypic characterization of Korean isolates of *Cronobacter* spp. (Enterobacter sakazakii). J Microbiol Biotechnol 21: 509–514. 21617348 10.4014/jmb.1007.07059

[39] Mullane NR, Whyte P, Wall PG, Quinn T, Fanning S (2007) Application of pulsed-field gel electrophoresis to characterise and trace the prevalence of *Enterobacter sakazakii* in an infant formula processing facility. Int J Food Microbiol 116: 73–81 17307267 10.1016/j.ijfoodmicro.2006.12.036

[40] Proudy I, Bougle D, Coton E, Coton M, Leclercq R, Vergnaud M (2008) Genotypic characterization of *Enterobacter sakazakii* isolates by PFGE, BOX-PCR and sequencing of the fliC gene. J Appl Microbiol 104: 26–34. 17850301 10.1111/j.1365-2672.2007.03526.x

[41] Miled-Bennour R, Ells TC, Pagotto FJ, Farber JM, Kerouanton A, Meheut T, et al (2010) Genotypic and phenotypic characterisation of a collection of *Cronobacter* (*Enterobacter sakazakii*) isolates. Int J Food Microbiol 139: 116–125. 10.1016/j.ijfoodmicro.2010.01.045 20181403

[42] Lee YD, Ryu TW, Chang HI, Park JH (2010) Identification and Classification of *Cronobacter* spp. Isolated from Powdered Food in Korea. J Microbiol Biotechnol 4: 757–762 20467249

[43] Nazarowec-White M, Farber JM (1999) Phenotypic and genotypic typing of food and clinical isolates of *Enterobacter sakazakii* . J Med Microbiol 48: 559–567. 10359305 10.1099/00222615-48-6-559

[44] Stock I, Wiedemann B (2002) Natural antibiotic susceptibility of *Enterobacter amnigenus*, *Enterobacter cancerogenus*, *Enterobacter gergoviae* and *Enterobacter sakazakii* strains. Clin Microbiol Infect 8: 564–578. 12427217 10.1046/j.1469-0691.2002.00413.x

